# Immune Status, Strain Background, and Anatomic Site of Inoculation Affect Mouse Papillomavirus (MmuPV1) Induction of Exophytic Papillomas or Endophytic Trichoblastomas

**DOI:** 10.1371/journal.pone.0113582

**Published:** 2014-12-04

**Authors:** John P. Sundberg, Timothy M. Stearns, Joongho Joh, Mary Proctor, Arvind Ingle, Kathleen A. Silva, Soheil S. Dadras, A. Bennett Jenson, Shin-je Ghim

**Affiliations:** 1 The Jackson Laboratory, Bar Harbor, ME, United States of America; 2 James Graham Brown Cancer Center (JGBCC), School of Medicine, University of Louisville, Louisville, KY, United States of America; 3 Tata Memorial Centre, Kharghar, Navi Mumbai, India; 4 Dept. Dermatology and Genetics/Developmental Biology, University of Connecticut Health Center, Farmington, CT, United States of America; Georgetown University, United States of America

## Abstract

Papillomaviruses (PVs) induce papillomas, premalignant lesions, and carcinomas in a wide variety of species. PVs are classified first based on their host and tissue tropism and then their genomic diversities. A laboratory mouse papillomavirus, MmuPV1 (formerly MusPV), was horizontally transmitted within an inbred colony of NMRI-*Foxn1^nu^/Foxn1^nu^* (nude; T cell deficient) mice of an unknown period of time. A ground-up, filtered papilloma inoculum was not capable of infecting C57BL/6J wild-type mice; however, immunocompetent, alopecic, S/RV/Cri-*ba/ba* (bare) mice developed small papillomas at injection sites that regressed. NMRI-*Foxn1^nu^* and B6.Cg-*Foxn1^nu^*, but not NU/J-*Foxn1^nu^*, mice were susceptible to MmuPV1 infection. B6 congenic strains, but not other congenic strains carrying the same allelic mutations, lacking B- and T-cells, but not B-cells alone, were susceptible to infection, indicating that mouse strain and T-cell deficiency are critical to tumor formation. Lesions initially observed were exophytic papillomas around the muzzle, exophytic papillomas on the tail, and condylomas of the vaginal lining which could be induced by separate scarification or simultaneous scarification of MmuPV1 at all four sites. On the dorsal skin, locally invasive, poorly differentiated tumors developed with features similar to human trichoblastomas. Transcriptome analysis revealed significant differences between the normal skin in these anatomic sites and in papillomas versus trichoblastomas. The primarily dysregulated genes involved molecular pathways associated with cancer, cellular development, cellular growth and proliferation, cell morphology, and connective tissue development and function. Although trichoepitheliomas are benign, aggressive tumors, few of the genes commonly associated with basal cell carcinoma or squamous cells carcinoma were highly dysregulated.

## Introduction

Papillomaviruses (PVs) are small DNA viruses that induce exophytic, sessile, and endophytic papillomas (lay term: warts) as well as squamous cell carcinomas in most mammalian species including humans [1]. PVs are not only relatively species-specific, but also relatively anatomic site-restricted. Although new PV types are classified based on their degree of genetic relatedness to other known PVs, historically PVs were categorized as mucosotropic or cutaneotropic based on their tissue tropism. These characteristics hindered early development of a useful mouse model for human papillomavirus (HPV) infections. Mucosotropic HPVs cause virtually all cervical cancers, most anal cancers, and at least 25% of head and neck cancers [2]–[4]. The cutaneotropic HPVs, such as EV (epidermodysplasia verruciformis) type HPVs (HPV5 and 8), can cause cancers in genetically immunocompromized individuals [5] or serve as a cofactor inducing other skin cancers [6], [7]. UV light may also play a role in HPV-induced cancer [8], [9].

While much has been learned from studying papillomavirus infections in many domestic and wild mammalian species, primarily the bovine papillomaviruses (BPVs), cotton rabbit PV (CRPV), and canine PVs (COPV and CfPV2) [1], rodent PVs would be extremely valuable as mice are the premier animal model species for modeling human diseases [10]. Xenografts, a very artificial system, provided a means to propagate HPVs in immunodeficient rodents [11], [12], but this approach is not the same as a natural infection in a laboratory rodent. A number of papillomaviruses were discovered that naturally infected domestic, wild, and exotic rodent species (*Apodemus sylvaticus*, AsPV1 [13]; *Erethizon dorsatum*, EdPV1 [14]; *Mastomys natalensis*, MnPV1 and McPV2 [15]–[18]; *Mesocricetus auratus* papillomavirus, MaPV [19], [20]; *Micromys minutus*, MmiPV [21], [22]; *Miniopterus schreibersii*, MscPV1 [23]; *Mus musculus domesticus*, MmuPV1 [24]–[26], and the MmuPV1 variant [13]; *Peromyscus maniculatus,* PmPV type 1 [27]; *Rattus norvegicus*, RnPV1 and RnPV2 [13], [28]; and *Rousettus aegyptiacus*, RaPV [29]).

The first laboratory mouse papillomavirus was found to naturally infect NMRI-*Foxn1^nu^* nude mice that were T-cell deficient [24]. The virus was used to successfully infect bare mutant mice (S/RV/Cri-*ba/ba*) which supposedly has an intact immune system [30]. Based on the published histopathology, bare mutant mice are possibly due to mutations in the desmoglein 4 gene [31]– or another gene in the pathway that involves this gene. This new virus was considered to be a laboratory mouse papillomavirus, initially abbreviated MusPV, currently reclassified as MmuPV1 [13]. MmuPV1 induced classical papillomas with epidermal hyperplasia on thin fibrovascular stalks in a verrucous pattern [24]. The MmuPV1 genomic DNA is 7510 bp in size and contains at least seven open reading frames (ORFs; E1, E2, E4, E6, E7, L1, and L2), consistent with known PVs. Phylogenetic analysis revealed that MmuPV1 belongs to the π-genus together with 4 other rodent PVs (McPV2, MaPV1, MmiPV, and RnPV1) [25].

We report here that T-cell deficiency is critical for infection in a limited survey of mouse strains carrying a variety of single gene mutations that cause immunodeficiencies. B-cell deficiency alone is not permissive. There are strain specific differences in susceptibility suggesting major modifier genes are involved in determining whether mammals can be infected or not. MmuPV1 can be used to experimentally infect muzzle, dorsal trunk, tail, or vagina, and induce the types of lesions that develop from these infections into sessile plaques to exophytic papillomas, to locally invasive, poorly differentiated trichoblastomas. Transcriptome studies reveal differences between unaffected anatomic sites, as well as between the tumor types that were unique not involving genes dysregulated in basal and squamous carcinoma mouse model.

## Results

To investigate the infectivity of MmuPV1 and its capability of inducing benign or malignant proliferative skin diseases at different anatomical sites, experimental infections were carried out using selected inbred mouse strains and congenic strains carrying a variety of single gene mutations that cause various forms of immunodeficiency (**Table 1**). MmuPV1 can naturally be highly contagious among T-cell-deficient nude mice. Co-housing MmuPV1-infected NMRI-*Foxn1^nu^/Foxn1^nu^* (nude) mice with naïve NMRI-*Foxn1^nu^/Foxn1^nu^* mice was sufficient to infect the naïve mice. As illustrated and described in the original outbreak [24], affected tumor donor mice developed numerous small filiform papillomas on their face, neck, trunk, legs, and tail. Most lesions were 0.1 cm in diameter and 0.2 cm in height. Sessile to exophytic, verrucous papillomas on the tail were up to 0.4 cm×0.1 cm. Mucosa lesions were not observed in the oral cavity, conjunctiva, or genital areas at the time of necropsy in the spontaneous outbreak.

**Table 1:** Summary of tumor type induction at various anatomic sites by scarification with MusPV on different inbred/congenic mouse strain backgrounds carrying various mutations causing immunodeficiencies.

Deficiency	Strain/Mutation	Muzzle	Back	Tail
Normal	C57BL/6J +/+	No tumor	No tumor	No tumor
Normal, alopecia [24]	S/RV/Cri-*ba/ba*	Not Tested	Papilloma (?)	Not Tested
T cells	NU/J-*Foxn1^nu^*/J	No tumor	No tumor	Not Tested
T cells	B6.Cg-*Foxn1^nu^*/J	Papilloma	Trichoblastoma	Papilloma
T cells [24]	NMRI-*Foxn1^nu^/Foxn1^nu^*	Papilloma	Trichoblastoma	Papilloma
B cells	B6.129S2-*Ighm^tm1Cgn^*/J	No tumor	No tumor	No tumor
T & B cells	B6.129S7-*Rag1^tm1Mom^/*J	Papilloma	Trichoblastoma	Papilloma
T & B cells	B6.CB17-*Prkdc^scid^*/SzJ	Papilloma	Trichoblastoma	Not Tested
T & B cells	NOD.CB17-*Prkdc^scid^*/SzJ	No tumor	No tumor	No tumor
CD4^+^ T cells	B6.129S2-*Cd4^tm1Mak^*/J	No tumor	No tumor	No tumor
CD8^+^ T cells	B6.129S2-*Cd8^tm1Mak^*/J	No tumor	No tumor	No tumor

(?) No histology done because tumors spontaneously regressed [24].

**Figure 1:**
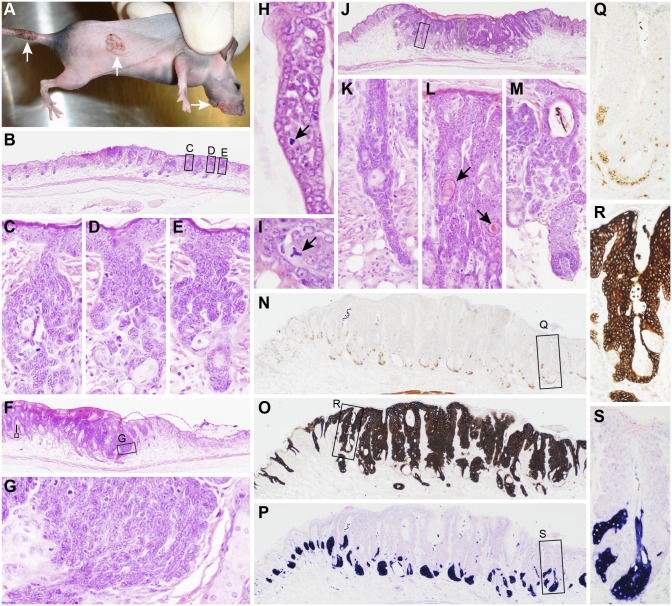
Cutaneous papillomas and trichoblastomas in mice. A representative B6.Cg-*Foxn1^nu^* ***/*** *Foxn1^nu^* (nude) mouse was scarified and inoculated with MmuPV1 on the muzzle, dorsal, and tail skin. Verrucous proliferations arose at each of the injection sites (A, arrows). The earliest lesions consisted of acanthosis, small focal ulcers, and medusiform proliferations of hair follicle root sheaths around and above the sebaceous glands (B–E). Slightly more advanced lesions had larger sheets of basaloid cells with hyperchormatic nuclei and a high mitotic index (F, G) occasionally with tripolar mitotic figures (arrows, H, I). Other invasive lesions (J) had long extensions from the base of hair follicles (K, enlargement of boxed area in J), proliferative areas with “keratin” pearl formations (arrows, L) or normal early-stage anagen follicles with medusiform proliferating root sheath cells in the bulge area resulting in concurrent follicular dystrophy (M). Sebaceous glands were unaffected as reserve cells were not expanded nor in abnormal locations when labeled for SOAT1 expression (N, Q, enlargement of boxed area in N), were completely outlined by the proliferating root sheath cells based on KRT14 expression (O, R, enlargement of boxed area in O), and mature sebocytes were organized in a normal proliferation pattern when labeled with adipophilin (P, S, enlargement of boxed area in P).

To determine if tumors were initially infectious, ground and clarified tumor suspensions were injected intradermally into four T-cell-deficient B6.Cg-*Foxn1^nu^/Foxn1^nu^* and four NU/J-*Foxn1^nu^/Foxn1^nu^* 8 week old female mice into the skin of the dorsal lumbar area. At 36 days post-inoculation, only the B6 nude mice had very small, white areas at the injection sites. These became slightly raised, erythematous, and very mildly ulcerated 43 days after injection at which time the mice were euthanized. No lesions were noted at the gross or histologic level in the NU/J-*Foxn1^nu^/Foxn1^nu^* nude mice. By contrast, the skin from the B6.Cg-*Foxn1^nu^/Foxn1^nu^* nude mice had mild erythematous crusty areas. This was repeated using scarification of the tail, dorsal skin above the paralumbar fossa of the trunk and facial skin. These mice developed what grossly appeared to be papillomas at the infection sites (**Fig. 1A**).

Following the initial study to determine infectivity, experimental inductions of skin tumors could be repeated using 0.3 ug of L1 protein per dose of inoculum at each site. B6.Cg-*Foxn1^nu^/Foxn1^nu^* mice were used as positive controls in each transmission study since these mice consistently developed lesions. Scarification was determined to be the method of choice and was used in all subsequent studies. Wild type C57BL/6J did not develop lesions at any of the inoculation sites in any of these studies. Proliferative lesions above the level of the skin were consistently observed in all susceptible mice in 3 to 9 weeks after inoculation with an average of 6 weeks (**Table 1**). In general, by 8 weeks after inoculation mice had to be euthanized as tumors reached 1 cm in diameter, the size limit imposed by the IACUC. Inoculation sites re-epithelialized within a few days after light scarification with no untoward effects.

The outcome of experimental MmuPV1 transmission studies at the three different anatomical sites in the selected mice strains are summarized in **Table 1**. C57BL/6J wild type mice developed antibodies against MmuPV1 but failed to develop tumors at any inoculation site, as reported previously [26]. By contrast, B6 congenic strains that lacked most or all T-cell subsets (nude, severe combined immunodeficiency, or *Rag1^tm1Mom^*) developed tumors while those lacking only CD4^+^ or CD8^+^ T-cells or only B-cells did not develop tumors. T-cell-deficient B6 congenic strains (*Foxn1^nu^*, nude, *Prkdc^scid^*, severe combined immunodeficiency, and *Rag1^tm1Mom^*, recombinase activating gene 1 null), developed tumors consistently. By contrast, congenic strains on other backgrounds (NOD.CB17 and NU/J), and carrying the same allelic mutations (T-cell-deficiencies) did not, indicating that strain-specific modifier genes affect susceptibility to infection not just the immune status of the host.

**Figure 2:**
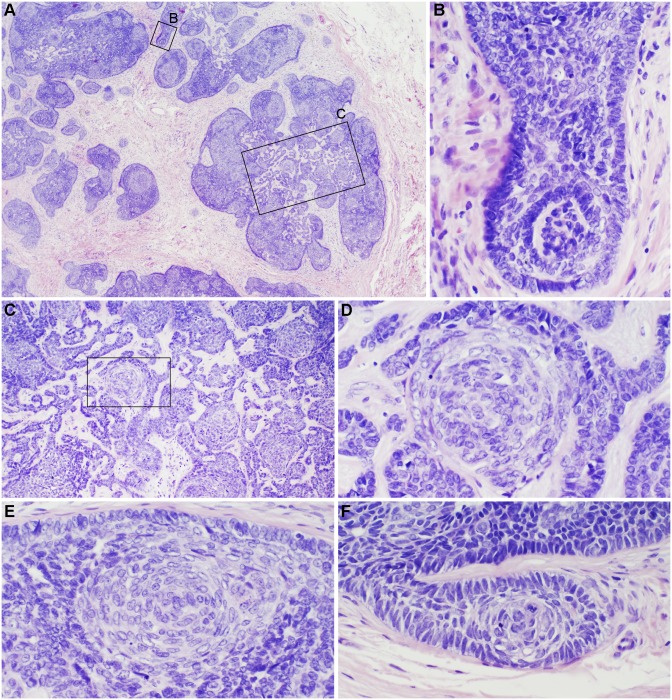
Human trichoblastoma. A trichoblastoma from a 68 year-old Caucasian male showed a pseudo-encapsulated, multinodular, basaloid tumor with fibrocellular stroma spanning the reticular dermis extending into subcutaneous fat (A). No epidermal connection or retraction artifact was noted. Tumor lobules were arranged as monomorphous basaloid cells in a cribriform pattern with peripheral palisading some resembling abortive hair follicles (B, F). Focally, tumor lobules exhibited squamous eddies, papillary mesenchymal bodies, and a germinative component comprising basaloid cells admixed with distinct pales cells (Zellballen) (C–E, D is an enlargement of boxed area in C).

Lesions from the dorsal skin of mice in the initial pilot study had moderate acanthosis with orthokeratotic hyperkeratosis of the epidermis. The region both above and below the bulge region (infundibulum and stem cell component of the hair follicle) of affected hair follicles within the inoculation site were markedly proliferative in a medusiform pattern somewhat reminiscent of a basal cell carcinoma (**Fig. 1B–M**). More extensive lesions expanded into the hypodermal fat layer as irregular sheets of cells with a high mitotic index, large nuclear to cytoplasmic ratio, and poorly defined borders (**Fig. 1G**). In other areas, finger-like extensions surrounded by what appeared to be linear arrangements of basal cells on the periphery and less organized cells in the center (**Fig. 1H, K**). Tripolar mitotic figures were found in the more invasive areas (**Fig. 1H, I**). Sebaceous glands appeared to be unaffected (**Fig. 1E, K**). Reserve cells, positive for sterol O-acyltransferase 1 (SOAT1) (**Fig. 1N, Q**), the entire sebaceous gland, outlined by keratin 14 (KRT14, **Fig. 1O, R**), and sebocytes, positive for adipophilin (PLIN2, perilipin 2**,** **Fig. 1P, S**) were normal. These changes were found consistently in lesions induced in the dorsal skin. Koilocytosis with immunohistochemically positive nuclei for papillomavirus group-specific antigens were present but in relatively low numbers. While the medusiform features resemble basal cell carcinomas (**Fig. 1E, M**), rare “keratin” pearls suggested squamous cell carcinomas (**Fig. 1L**). More solid, poorly differentiated areas could be described simply as locally invasive, poorly differentiated carcinomas (**Fig. 1G**). The pattern and site of development of these tumors, extending from the region of the bulge where the hair follicle stem cells are located, were more consistent with the features of a trichoblastoma as described in humans (**Fig. 2**) [34]–[37], dogs, cats, horses, and sheep [38]–[40].

**Figure 3:**
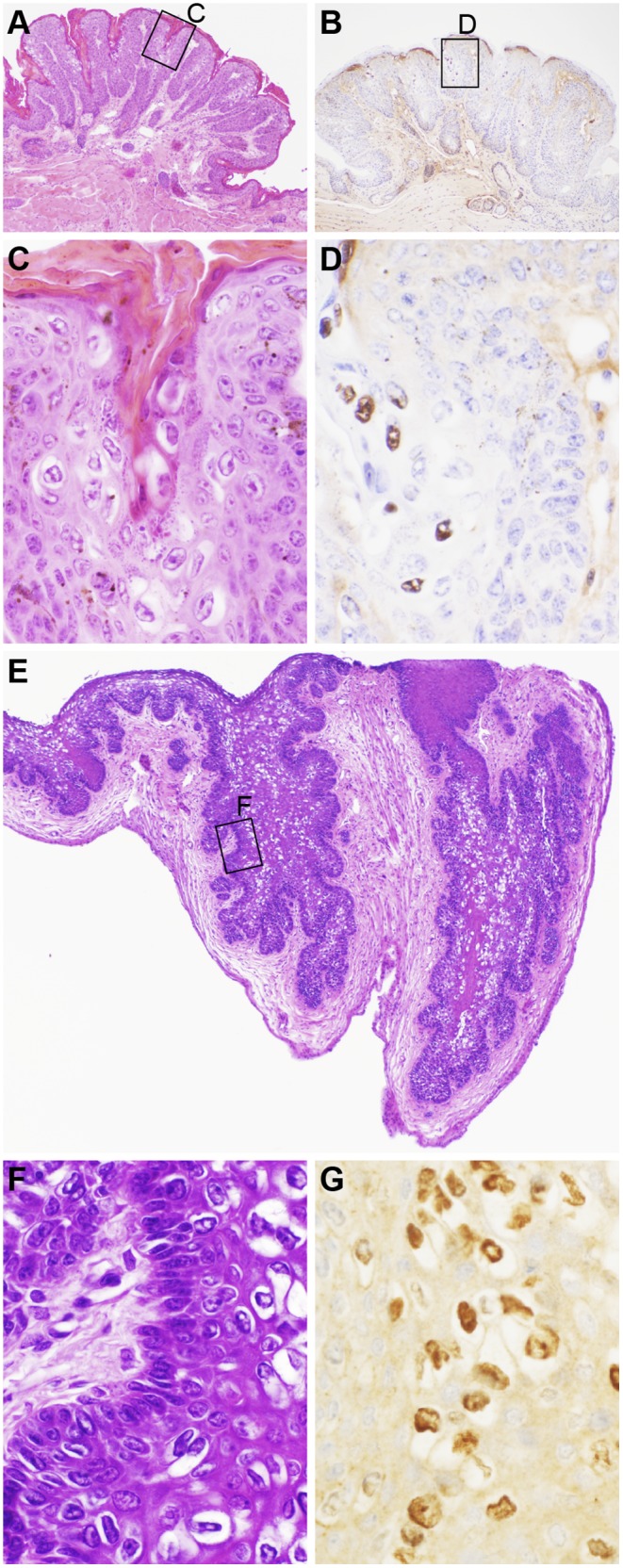
Papillomas from the mouse tail and vagina. Inoculation sites on the tail resulted in epithelial proliferations on thin fibrovascular stalks (A). Boxed area reveals koilocytes within the stratum granuloum (C). These koilocytes were positive for papillomavirus antigens by immunohistochemistry (B, D). The infected vagina had diffuse mucosal hyperplasia with irregular invasion into the surrounding wall (E). Boxed area is enlarged to illustrate the marked koilocytosis (F). The koilocytes in the vagina had positive expression of papillomavirus antigens by immunohistochemistry (G).

By contrast, lesions induced on the muzzle and tail skin were exophytic papillomas, similar to those seen in most mammals with papillomavirus infections (**Fig. 3A, C**). These lesions consisted of epithelial proliferation on thin fibrovascular stalks in a verrucous pattern raised above the level of the skin. Koilocytes were present in the stratum granulosum which were positive for papillomavirus group-specific antigens by immunohistochemistry (**Fig. 3B, D**).

The vagina was infected experimentally and all mice developed sessile to locally invasive and extensive mucosal proliferation associated with very high levels of koilocytosis. Koilocytes were positive for papillomavirus group-specific antigens (**Fig. 3E–G**).

**Figure 4:**
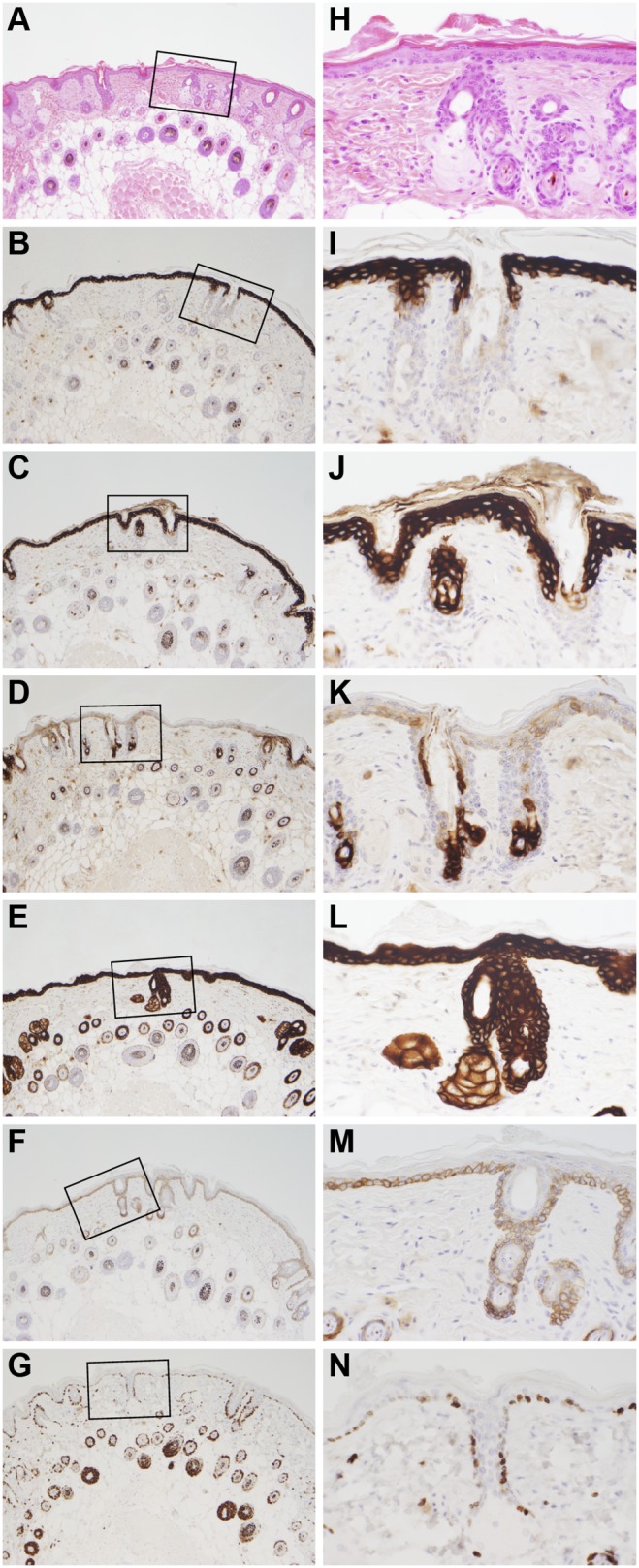
Immunohistochemical labeling of nude mouse skin. The B6.Cg-*Foxn1^nu^* nude mouse, like most mutant mice with alopecia, has mildly hyperplastic epidermis as seen in routine H&E stained sections (A, H). Terminal differentiation proteins (KRT1, B, I; and 10, C, J) are diffusely expressed above the stratum basale and below the stratum corneum. Keratin 6A (KRT6A) is normally expressed in the companion layer of the hair follicle but will be expressed higher in the follicle and in the epidermis during hyperplastic changes (D, K). Keratin 14 (E, L) and keratin 5 (F, M) label the basal cells. Keratin 14 is often more diffusely expressed as is the case here due to the mild acanthosis in nude mouse skin. Proliferating cells, as indicated by Ki67 labeling, is normally limited to the basal cells of the epidermis, outer root sheath of the hair follicle, and especially in anagen hair bulbs (G, N).

**Figure 5:**
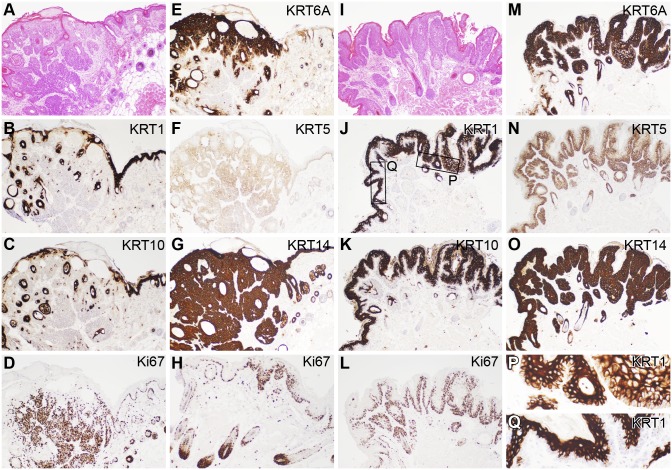
Immunohistochemical comparison of mouse trichoblastoma and papilloma. Trichoblastomas exhibited both epidermal proliferation and irregular invasion into the dermis and hypodermal fat layers (A, H&E stain) in comparison to papillomas that were exophytic (I, H&E stain). KRT1 and 10, normally expressed in the suprabasal epidermis, were downregulated in the trichoblastomas (B, C) but upregulated in the papillomas (J, K). However, in papillomas, while these keratins were suprabasalar in expression in normal and hyperplastic epidermis, their expression was throughout the epidermis in the papillomas (P, Q). KRT5 and 14, normally found primarily in basal cells, were diffusely upregulated in the trichoblastomas (F, G) as well as the papillomas (N, O), although KRT5 was more localized to expressed in basal cells of papillomas. Ki67, a marker of cell proliferation, was diffuse in the invading trichoblastoma (D) in sharp contrast to the junction of the trichoblastoma and hyperplastic epidermis (H) or papillomas (L) where expression was primarily restricted to basal cells and anagen bulbs.

Immunohistochemical analysis of the mildly hyperplastic “normal” skin of the nude mouse (**Fig. 4**), compared to trichoblastomas (**Fig. 5A–H**) and papillomas (**Fig. 5I–Q**), revealed similar patterns of protein expression for KRT1 and 10 which are normally expressed in the suprabasilar epidermis. Expression was downregulated in the trichoblastomas (**Fig. 5B, C**) but upregulated in the papillomas (**Fig. 5J, K**). In the papillomas, KRT1 was suprabasilar in hyperplastic epidermis adjacent to the papilloma (**Fig. 5Q**) but was expressed in all layers in the papillomatous areas (**Fig. 5P**). Keratins 5 and 14 are normally expressed in the basal cells with some dilution of expression in the suprabasal cells of normal skin. Keratin 14 was diffusely expressed in the epidermis and hair follicles in the areas of hyperplasia and throughout both papillomas and trichoblastomas (**Fig. 5G, O**). Keratin 5 was diffusely expressed in the trichoblastomas but primarily in the basal cells in papillomas (**Fig. 5F, N**). Proliferating cells were primarily in the stratum basale and hair follicle bulbs in normal to mildly hyperplastic areas of skin, but abruptly became basilar and suprabasilar at the edge of the trichoblastomas (**Fig. 5H**). The pattern of proliferation was very different between trichoblastomas, which had very high Ki67 expression in the locally invading areas (**Fig. 5D**), in comparison to the papillomas where this was restricted primarily to the stratum basale (**Fig. 5L**).

### Viral genomic detection in lesions

In order to determine if the virus was in any or all of the tumors, all lesions were collected and processed for DNA screening by PCR methods. The presence of MmuPV1 DNA was confirmed from all papillomas (both exophytic and sessile) and trichoepitheliomas (data not shown), as was demonstrated in the natural outbreak and early transmission studies [24], [26].

### Transcriptome analyses

**Figure 6:**
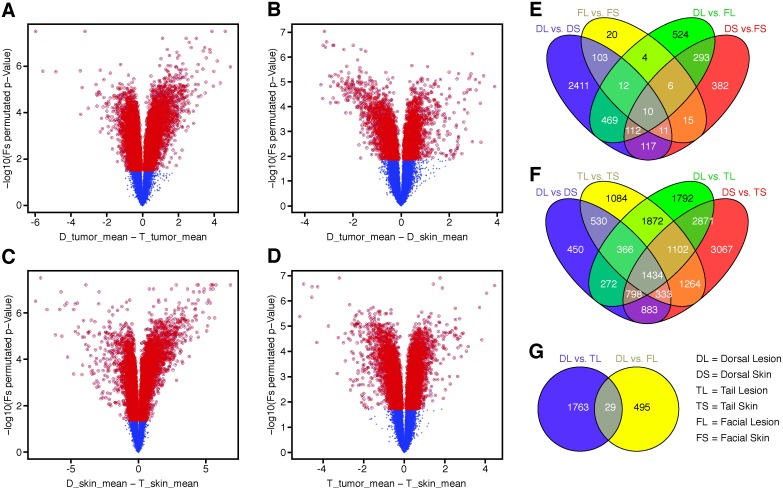
Transcriptome analyses. Volcano plots of gene array comparisons between dorsal skin trichoblastoma and tail skin papilloma (A), Dorsal Skin Carcinoma vs. Normal Dorsal Skin (B), Normal Dorsal Skin vs. Normal Tail Skin (C), and Tail Papilloma vs. Normal Tail Skin (D) reveal large numbers of significantly dysregulated probe sets (red dots) in all comparisons. Venn diagram illustrates the numbers of probe sets significantly dysregulated (q-value<0.05) when comparing the dorsal trichoblastoma and tail papilloma with normal tissues from the same sites (E) or dorsal trichoblastoma and facial papilloma with normal tissues (F). Twenty-nine common probe sets (26 genes) were uniquely dysregulated when papillomas from either the face or tail were compared to the dorsal trichoblastoma (G). DS, dorsal skin; DL, dorsal lesion; FS, facial skin; FT, facial lesion; TS, tail skin; TL, tail lesion.

Two separate gene array experiments were done at two institutions, The Jackson Laboratory and University of Louisville, concurrently. One compared dorsal skin lesion, trichoblastomas, with tail skin papillomas and the respective normal skin. The second compared the dorsal skin lesions to papillomas on the face and the respective normal skin. Transcriptome analyses revealed large numbers of probe sets that were significantly dysregulated (q-value<0.05) when comparing dorsal trichoblastomas to tail papillomas (**Fig. 6A**), dorsal trichoblastomas to normal dorsal skin (**Fig. 6B**), normal dorsal skin to normal tail skin (**Fig. 6C**), and tail papillomas to normal tail skin (**Fig. 6D**). While it was anticipated that there would be big differences between tumor types or the tumors and control skin from the same location, there were comparable degree changes between the normal dorsal and tail skin. This was more evident using Venn diagrams with the numbers of dysregulated probe sets in each comparison (**Fig. 6E–G**). To dissect the most significant candidate genes involved in the pathogenesis of the tumors, 26 dysregulated genes (identified by 29 dysregulated probe sets) were identified when compared between dorsal lesions and tail lesions and between dorsal lesions and facial lesions (**Table 2**). A number of genes have been associated with skin cancer, primarily basal cell carcinomas, using genetically engineered mouse models [41], [42]. While many, but not all, of these were found to be dysregulated (**Table 3**), even though significantly dysregulated, the fold change was not high. Changes did correlate with the protein expression observed by immunohistochemistry (**Fig. 5**).

**Table 2:** Summary of transcripts (29 probe sets for 26 genes) dysregulated in the overlap of comparisons between dorsal trichoblastomas versus tail papillomas and dorsal trichoblastomas and facial papillomas.

AffymetrixID	*DL vs. TL	p-value	q-value	*DL vs. FL	p-value	q-value	IPA ID	*Gene*	Description	Location	Family
10353028	1.2142	0.0003	0.0025	1.2483	0.0005	0.0214	10353028	*Vcpip1*	valosin containing protein (p97)/p47 complex interacting protein 1	Cytoplasm	peptidase
10360720	−1.1487	0.0123	0.0267	−1.217	0.0018	0.0391	10360720	*Wdr26*	WD repeat domain 26	Cytoplasm	other
10364038	1.2426	0.0164	0.033	1.4241	0.0013	0.0337	10364038	*Upb1*	ureidopropionase, beta	Cytoplasm	enzyme
10368199	−1.4208	0.0177	0.0349	−2.2868	0.0005	0.0214	10368199	*Myb*	v-myb myeloblastosis viral oncogene homolog (avian)	Nucleus	transcription regulator
10421810	1.2775	0.0031	0.0102	1.3348	0.0024	0.0455	10421810	*Rgcc*	regulator of cell cycle	Cytoplasm	other
10434806	1.192	0.0038	0.0117	1.3013	0.0023	0.0443	10434806	*Lpp*	LIM domain containing preferred translocation partner in lipoma	Nucleus	other
10436451	−1.3819	0.002	0.0075	−1.8319	0.0008	0.0274	10436451	*Stx19*	syntaxin 19	Other	other
10436967	1.2058	0.0163	0.0329	1.257	0.0024	0.045	10436967	*Cbr1*	carbonyl reductase 1	Cytoplasm	enzyme
10443391	−1.1728	0.0106	0.0241	−1.3165	0.0027	0.0478	10443391	*Mapk14*	mitogen-activated protein kinase 14	Cytoplasm	kinase
10445574	1.3044	0.0014	0.0061	1.434	0.0025	0.0462	10445574	*Cul7*	cullin 7	Cytoplasm	other
10454286	−1.8277	0.0003	0.0026	−1.6974	0.0008	0.027	10454286	*Mapre2*	microtubule-associated protein, RP/EB family, member 2	Cytoplasm	other
10455395	−1.4012	0.0011	0.0051	−1.5692	0.0018	0.04	10455395	*Spink5*	serine peptidase inhibitor, Kazal type 5	Extracellular Space	other
10489092	1.2599	0.001	0.005	1.2368	0.0018	0.0401	10489092	*Soga1*	suppressor of glucose, autophagy associated 1	Extracellular Space	other
10489831	1.1783	0.0003	0.0028	1.1251	0.0016	0.0374	10489831	*Stau1*	staufen, RNA binding protein, homolog 1 (Drosophila)	Cytoplasm	transporter
10491272	−1.2368	0.0285	0.0493	−1.4175	0.0009	0.0282	10491272	*Gpr160*	G protein-coupled receptor 160	Plasma Membrane	G-protein coupled receptor
10495001	1.162	0.0102	0.0234	1.2775	0.001	0.0297	10495001	*Rsbn1*	round spermatid basic protein 1	Nucleus	other
10500122	−1.1594	0.0132	0.0281	−1.5018	0.0004	0.0196	10500122	*Bnipl*	BCL2/adenovirus E1B 19 kD interacting protein like	Cytoplasm	other
10530819	−1.5511	0.0007	0.004	−1.7695	0.0016	0.0375	10530819	*Hopx*	HOP homeobox	Nucleus	transcription regulator
10531323	1.1408	0.0179	0.0353	1.2426	0.0019	0.0414	10531323	*G3bp2*	GTPase activating protein (SH3 domain) binding protein 2	Nucleus	enzyme
10537026	−1.234	0.0186	0.0362	−2.5491	0.0001	0.0093	10537026	*Cpa4*	carboxypeptidase A4	Extracellular Space	peptidase
10561837	1.1674	0.0158	0.0322	1.2687	0.0016	0.0374	10561837	*Znf146*	zinc finger protein 146	Nucleus	other
10569923	−1.1647	0.0133	0.0282	−1.6133	0.0009	0.028	10569923	*Lrrc8e*	leucine rich repeat containing 8 family, member E	Other	other
10573803	1.217	0.0122	0.0266	1.4573	0.0027	0.0477	10573803	*Cyld*	cylindromatosis (turban tumor syndrome)	Nucleus	transcription regulator
10583647	−1.1434	0.0071	0.018	−1.2512	0.0008	0.0266	10583647	*Dnm2*	dynamin 2	Plasma Membrane	enzyme
10595856	1.2002	0.0191	0.0369	1.4473	0.0022	0.0434	10595856	*Slc25a36*	solute carrier family 25 (pyrimidine nucleotide carrier), member 36	Cytoplasm	transporter
10597162	−1.1947	0.0149	0.0307	−1.4012	0.0008	0.0265	10597162	*Klhl18*	kelch-like family member 18	Other	other

Several oncogenes and cell cycle regulatory genes are identified. However, while statistically significant, fold changes in all cases are relatively minor suggesting a great deal of interaction may be necessary for viral induced tumors to differentiate into benign and productive papillomas rather than locally aggressive cancers. (*Fold changes; p-value and q-value<0.05).

**Table 3:** Many transcripts are commonly associated with carcinomas in two stage chemical carcinogenesis and UV light induced cutaneous cancers.

Symbol	Entrez Gene Name	Entrez	Affymetrix	Location	Family	DL vs.FL	DLvs. FL	DL vs.TL	DLvs. TL	DL vs.DS	DLvs. DS	TL vs.TS	TL vs.TS	DS vs.TS	DS vs.TS
		Gene ID	ID			(FoldChange)	(p-value)	(FoldChange)	(p-value)	(FoldChange)	(p-value)	(FoldChange)	(p-value)	(FoldChange)	(p-value)
*Nt5e*	5′-nucleotidase,ecto (CD73)	23959	10587639	PlasmaMembrane	Phosphatase	1.1701	0.2472	2.6329	0.0011	1.2658	0.0578	−2.0515	0.0024	1.0140	0.3742
*Krt15*	keratin 15	16665	10391025	Cytoplasm	Other	−1.0281	0.6567	2.5669	0.0015	−1.1147	0.3017	−1.8877	0.0041	1.5157	0.0078
*Sox4*	SRY (sexdeterminingregion Y)-box 4	20677	10408450	Nucleus	transcriptionregulator	1.2687	0.2394	1.8575	0.0022	−1.1540	0.1892	−1.7013	0.0043	1.2599	0.0353
*Trp53*	tumorprotein p53	22059	10377550	Nucleus	transcriptionregulator	1.0968	0.2757	1.3566	0.0025	1.1728	0.0347	−1.0070	0.5749	1.1487	0.0241
*Lgr5*	leucine-richrepeatcontainingG protein-coupledreceptor 5	14160	10372503	PlasmaMembrane	G-proteincoupledreceptor	−1.1594	0.4595	2.7574	0.0036	−1.2255	0.2888	−2.9485	0.0045	1.1460	0.2565
*Cd34*	CD34 molecule	12490	10352905	PlasmaMembrane	Other	−2.1189	0.0389	1.5440	0.0046	−2.0705	0.0042	−1.6702	0.0044	1.9141	0.0012
*Ptch1*	patched 1	19206	10410039	PlasmaMembrane	transmembrane receptor	−1.3044	0.3296	1.6021	0.0072	−2.3080	0.0048	−1.9634	0.0039	1.8834	0.0022
*Bnc2*	basonuclin 2	242509	10514177	Nucleus	Other	−1.0070	0.6822	2.0849	0.0078	−1.2198	0.2774	−2.6820	0.0047	−1.0546	0.3455
*Trp63*	tumor protein p63	22061	10434815	Nucleus	transcriptionregulator	1.1355	0.1975	1.1728	0.0103	1.2170	0.0120	−1.1434	0.0271	−1.1865	0.0063
*Krt1*	keratin 1	16678	10432852	Cytoplasm	Other	−1.2894	0.0234	−1.1947	0.0140	1.1173	0.1045	1.2058	0.0165	−1.1070	0.0670
*Ncdn*	neurochondrin	26562	10516427	Cytoplasm	Other	−1.0163	0.6246	−1.1810	0.0144	1.1225	0.0825	1.1045	0.1074	−1.2002	0.0081
*Krt10*	keratin 10	16661	10390831	Cytoplasm	Other	−1.2658	0.0669	−1.1865	0.0262	−1.1173	0.1365	−1.0842	0.2350	−1.1514	0.0393
*Ctnnb1*	catenin(cadherin-associatedprotein), beta1, 88 kDa	12387	10590325	Nucleus	transcription regulator	−1.0425	0.4810	1.1460	0.0311	1.0619	0.2507	−1.1225	0.0737	−1.0401	0.2360
*Lgr6*	leucine-richrepeatcontaining G protein-coupledreceptor 6	329252	10357965	PlasmaMembrane	G-proteincoupledreceptor	−1.2368	0.3612	1.3629	0.0369	−1.2894	0.1030	−1.6818	0.0085	1.0449	0.3280
*Gli2*	GLI family zinc finger 2	14633	10357137	Nucleus	transcriptionregulator	1.0000	0.6887	1.3629	0.0374	−1.2340	0.1518	−1.2687	0.1116	1.3256	0.0398
*Bmp6*	bonemorphogenetic protein6	12161	10404686	Extracellular Space	growth factor	−1.7371	0.0420	−1.2628	0.0438	−1.1620	0.1777	−1.1460	0.2147	−1.2454	0.0416
*Gli1*	GLI family zinc finger 1	14632	10373179	Nucleus	transcriptionregulator	−1.2805	0.2916	1.3256	0.0487	−2.1140	0.0062	−1.7451	0.0067	1.6058	0.0061
*Sox2*	SRY (sexdeterminingregion Y)-box2	20674	10491477	Nucleus	transcriptionregulator	−1.2340	0.5268	−1.1251	0.0605	−1.0767	0.2125	1.0353	0.4382	−1.0093	0.3707
*Hdgfrp3*	hepatoma-derivedgrowth factor,related protein 3	29877	10565193	Nucleus	Other	1.0918	0.2822	−1.1728	0.0661	1.0187	0.4871	−1.1810	0.0808	−1.4109	0.0037
*Krt75*	keratin 75	109052	10432746	Cytoplasm	Other	−2.7384	0.1060	2.0186	0.0867	2.0467	0.1221	−3.1095	0.0269	−3.1529	0.0146
*Lor*	loricrin	16939	10499870	Cytoplasm	Other	−1.3787	0.0614	1.1251	0.0896	−1.1810	0.0569	−1.4473	0.0038	−1.0892	0.1298
*Krt71*	keratin 71	56735	10432799	Cytoplasm	Other	−3.4983	0.2131	2.3187	0.1018	4.2281	0.0370	−3.3714	0.0502	−6.1475	0.0068
*Hras*	v-Ha-rasHarvey ratsarcoma viraloncogenehomolog	15461	10569071	PlasmaMembrane	Enzyme	−1.0473	0.5414	−1.0943	0.1195	1.0644	0.2593	−1.0497	0.3619	−1.2226	0.0078
*Smo*	smoothened,frizzled familyreceptor	319757	10536917	PlasmaMembrane	G-proteincoupledreceptor	1.0792	0.2169	1.1045	0.1913	1.2170	0.0695	1.0595	0.4119	−1.0401	0.2981
*Gli3*	GLI familyzinc finger 3	14634	10403727	Nucleus	transcriptionregulator	1.4540	0.0711	1.1514	0.2241	1.0093	0.5282	−1.6935	0.0097	−1.4845	0.0150
*Sufu*	suppressor offused homolog(Drosophila)	24069	10463645	Nucleus	transcriptionregulator	−1.0473	0.4255	1.0842	0.2256	1.1355	0.1527	1.1487	0.1235	1.0968	0.1464
*Shh*	sonichedgehog	20423	10528872	Extracellular Space	Peptidase	−1.0546	0.6215	1.1594	0.2292	−1.4845	0.0438	−1.1892	0.2531	1.4473	0.0240
*Lphn1*	latrophilin 1	330814	10580056	PlasmaMembrane	G-proteincoupledreceptor	−1.0968	0.3626	1.0595	0.2629	−1.0449	0.3645	−1.1865	0.0375	−1.1947	0.0208
*Pax6*	paired box 6	18508	10474312	Nucleus	transcriptionregulator	−1.0595	0.5476	−1.0497	0.3166	1.0546	0.2649	1.0353	0.4101	−1.0619	0.1439
*Krt14*	keratin 14	16664	10391052	Cytoplasm	Other	1.0867	0.5098	−1.0210	0.3967	1.4340	0.0042	1.0892	0.1294	−1.3441	0.0014
*Krt17*	keratin 17	16667	10391066	Cytoplasm	Other	−1.1019	0.3117	1.0140	0.4610	1.1460	0.1135	1.5122	0.0035	1.3379	0.0046

Many had no significant changes, and those that did, in general, the changes were not high. Some of the keratins are downregulated in the dorsal skin trichoblastomas compared to the tail and facial skin papillomas which corresponds to the immunohistochemical results.

**Figure 7:**
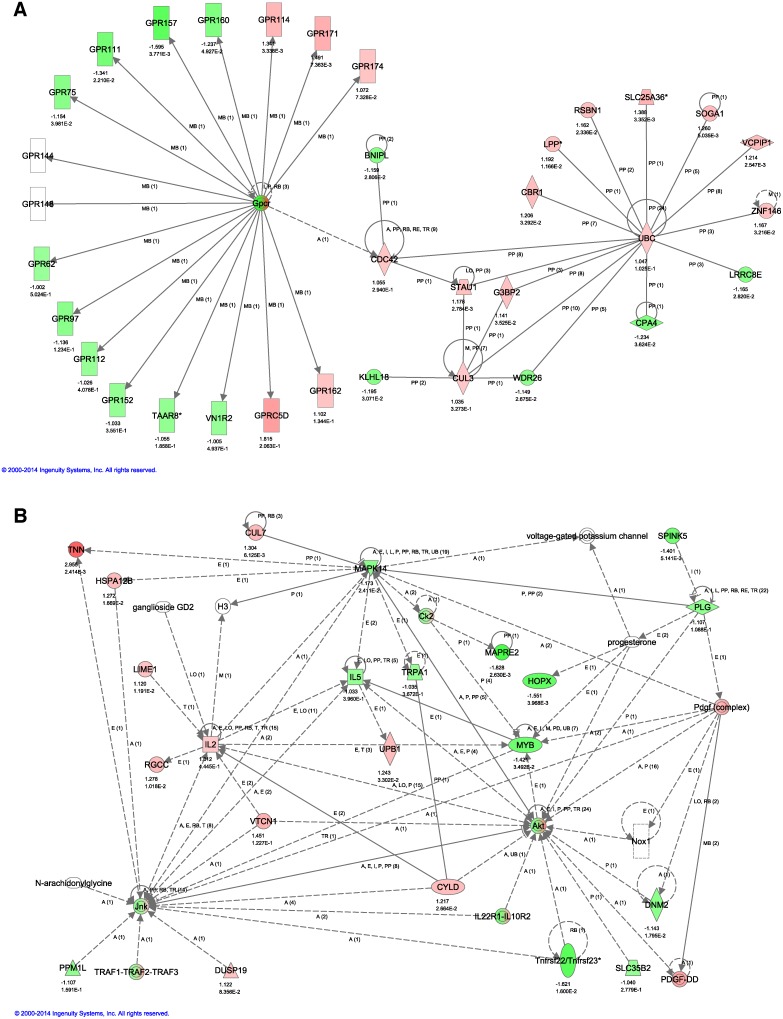
Dysregulated gene networks. Twenty-nine probe sets (representing 26 genes) were identified as uniquely dysregulated in the differential expression analyses of dorsal versus tail lesions and dorsal versus facial lesions. These 26 genes were used as input in Ingenuity's Pathway Analysis tool. This tool generates networks by attempting to connect submitted genes of interest together using Ingenuity’s Knowledge Base constructed from curated literature. The cell morphology and connective tissue-associated cancer network (A) and the cellular development and growth-associated cancer network (B) are (statistically) significantly associated with the function of cancer. Red is upregulation while green is downregulation.

The 26 dysregulated genes were used as input in Ingenuity's Pathway Analysis tool. This tool generates networks by attempting to connect submitted genes of interest together using Ingenuity’s Knowledge Base constructed from curated literature. Two networks were identified, the cell morphology and connective tissue-associated cancer network (**Fig. 7A**) and the cellular development and growth-associated cancer network (**Fig. 7B**). Both networks are significantly associated with the function of cancer. In the cell morphology and connective tissue-associated cancer network (**Fig. 7A**), *Ubc* (ubiquitin C) is directly connected to all but three genes of interest (*Bnipl*, *Gpr160*, and *Klhl8*). Ubiquitination is a post-translational modification that can serve multiple purposes including clearing out of harmful viral proteins, altering cellular location, and promote or prevent protein interactions [43]–[45]. The ubiquitin-proteasome pathway plays a critical role in protein turnover in cell cycle regulation; therefore, disruption of genes involved in this process can be the result of oncogenic mutations [46]. Nine of the 12 genes of interest directly connected to *Ubc* are upregulated in malignant tissue. Overexpression of several of these genes has been previously identified in various cancer types: *Slc25a36* (cervical) and *Znf146* (colorectal and pancreas) [47], [48]. Additionally, dysregulation of *Vcpip1* has been identified as mutated in 2% of cancers, including large intestine, skin, and urinary tract [49]. Several genes of interest are also shown as downregulated, such as, *Bnipl*, whose expression has been shown to be reduced in breast cancers [50]. The presence and preservation of expression change of these documented genes in this network may help us identify potential cause(s) of location-specific malignancy.

The cellular development and growth-associated cancer network (**Fig. 7B**) contains a mix of both up and downregulated genes of interest. *Cul7* is a novel anti-apoptotic gene whose overexpression has been associated with non-small cell lung carcinoma [51]. Additionally, *Rgcc* has been identified to be overexpressed in numerous cancer types including urinary bladder, breast, colon, lung, prostate, and ovary [52]. Alternatively, deletion and silencing of several genes of interest, *Mapk14*, *Hopx,* and *Spink5*, have been associated with several tumor types, as well. Human lung tumors show reduced expression levels of MAPK14, and tumor abundance, formation, and progression are all increased in *Mapk14*-null mice expressing oncogenic *Kras* [53]. Epigenetic gene silencing of *Hopx* has been shown to promote progression in colorectal cancer [54]. Deletion of chromosome arm 5q, which contains *Spink5*, has been reported in human esophageal cancer [55]. Furthermore, *Spink5* has been identified as the gene causing Netherton’s Syndrome, and there are case reports of patients with this disease also having papillomatous skin lesions and cutaneous malignancies [56], [57]. HPV DNA has been detected in a number of lesions from such affected individuals [56], [57]. Given the presence of these well-documented, cancer-related genes, this network may also provide additional information into how location-specific malignancy is occurring. Failure to identify one or more highly dysregulated pathway(s), especially related to those described for induced skin cancers indicates that this model system is either very complicated and different or that the multiple site analysis approach is too complicated for currently available analytical tools.

## Discussion

Since our initial and subsequent reporting of MmuPV1 experimentally infecting mice [24]–[26], there have been a number of other reports on MmuPV1. The MmuPV1 variant (HQ625439) was isolated from normal ear skin from a wild, non-laboratory *Mus musculus* [13]. Pseudoviruses were also developed from our molecular characterization of MmuPV1 and were used to transduce BALB/c mouse skin at sites of trauma visualized using a luciferase reporter gene and reported that both mouse skin and genital tissues were permissive for this virus to cause infection [58]. Lesions were not observed in either report. However, these reports confirm that mice do carry their own papillomavirus in the wild and it can infect, under the right circumstances, laboratory mice. The studies using pseudovirus also confirm that both haired skin and genital tissues can potentially become infected by this virus. Others synthesized the MmuPV1 genome and were able to reproduce infection including malignant potential [59], [60]. The current report confirms these recent findings, defines the lesions that appear to have malignant potential as being similar to human trichoblastomas, demonstrate the role of the immune system and strain background, and reveals the complexity of the molecular pathways involved with infection and tumor induction.

MmuPV1 is the only PV isolated from a laboratory mouse colony that is capable of infecting laboratory mice experimentally. However, initial studies indicated that only immunodeficient mice were susceptible to infection resulting in tumor formation [24]. By infecting a panel of mouse strains that were immunocompetent or had various T-cell, B-cell or combined immunodeficiencies, it became evident that pan T-cell but not B-cell or specific T-cell subset deficiencies were necessary for mice to become infected by MmuPV1 and develop tumors.

Finding papillomavirus group-specific antigens in MmuPV1 papillomas within the hair follicle infundibulum was first described in cats [61], [62]. Prominent koilocytosis in the infundibulum implies that this is a preferential site for infection in haired skin. The earliest lesion on the dorsal skin in these mice (medusiform proliferation) arises above and around the level of the sebaceous gland, and below the level of the interfollicular epidermis, also indicates that the infundibulum is the target region. Early lesions associated with cutaneous two-stage chemical carcinogenesis in mice also arise in this area [63]–[65]. Papillomaviruses are well known to target biological transition zones, most notably the transition zone in the human cervix where the epithelial cell type changes [66], [67]. In the skin the biological transition zone is in the infundibulum where the interfollicular epidermis changes to the hair follicle epithelium. This extends to the level of the sebaceous gland duct where there is a physical barrier in the form of the inner root sheath [68]. The virus can infect this area and sebum extrusion can act as an organic solvent to potentially exacerbate other effects on the viral infection [69]. One of the values of a mouse model is that genetically engineered mice with various reporter genes linked to the hair follicles can be crossed with immunodeficient mice to clearly define which cell types are affected.

The number of normal dysregulated genes compared between the dorsal skin versus tail skin were almost 10 times more numerous than a similar comparison between normal dorsal skin and normal facial skin. This may be due to the fact that tail skin is anatomically much different from the facial and dorsal skin. Tail skin has a unique hair type which is much larger in diameter but shorter in length than the pelage hair. Body and facial hair have the 4 major hair types (none found on the tail). The facial skin has vibrissae which are also found above the feet. The epidermis of tail skin is very thick compared to dorsal and facial skin. There are other differences which make the tail skin very different from dorsal and facial skin but dorsal and facial skin are very similar to each other except for the vibrissae [68], [70]. These analyses reveal the complexity of these seemingly similar anatomic structures and the reason why viruses can have very specific tropisms and why lesions may differ in different locations.

While our investigations of other types of spontaneous mouse cancers revealed clear cut molecular pathways with major dysregulation of transcripts leading to novel insights into the pathogenesis [71], that was not the case here. Of the overlapping dysregulated transcripts between the benign facial and tail lesions compared with the aggressive back lesions revealed changes in oncogenesis (*Myb*), cell cycle (*Rgcc*), and apocrine sweat gland tumors (*Cyld*) the relative fold changes were not large. Of the 26 dysregulated genes in this group, only two had a fold change of greater than two (*Myb*, −2.2868; *Cpa4*, −2.5491). Null mutations induced in the myeloblastosis oncogene (*Myb*) in genetically engineered mice are associated with severe hematopoietic abnormalities (www.informatics.jax.org, 18 Feb 2014). Null mutations for carboxypeptidase 4 (*Cpa4*) have yet to be created from targeted embryonic stem cells (www.informatics.jax.org, 18 Feb 2014); however, *CPA4* polymorphisms in humans are associated with early onset intermediate to high risk prostate cancer [72].

Of the groups of genes often associated with squamous cell and basal cell carcinomas in UV light and two-stage chemical carcinogenesis protocols or genetically engineered mouse models, many were significantly dysregulated (q-value<0.05) but very few had relative fold changes greater than 2.0. Those with the greatest change, keratins 71 and 75 (*Krt71* and *Krt75*; formerly *K6hf*) are associated with the companion layer of the hair follicle and when mutated result in hair structural abnormalities [73]–[75]. Earlier studies, using a rabbit polyclonal antibody against what was then referred to as keratin 6, defined normal expression in the companion layer of the hair follicle. This expression pattern changed to include overexpression in hyperplastic and neoplastic interfollicular epidermis due to any of a number of causes [64], [76], [77]. Therefore, it was not surprising to see these changes in the tissues sections using immunohistochemistry. Keratins 1 and 10 (*Krt1* and *Krt10*), suprabasilar terminal differentiation proteins, were downregulated in the tumors. This expression pattern change corresponds to previous immunohistochemistry-based studies of skin tumor induction by other means in mice [64], [69] and the concept that cancer is a failure of cells to normally undergo terminal differentiation. Keratin 15 (*Krt15*) is associated with the hair follicle stem cells in the bulge region [78], which are the purported targets of cutaneous carcinogenesis [79], so it is not surprising to see *Krt15* being upregulated when comparing the aggressive dorsal lesions with tail lesions. However, another stem cell marker associated with skin cancer, *Cd34* [80], was upregulated comparing dorsal and tail normal skin but downregulated when lesions were compared to normal or the aggressive dorsal lesion to the more benign tail lesion. Another gene associated with hair follicle stem cells, *Lgr5*, was significantly upregulated when dorsal and tail lesions were compared but down regulated when dorsal lesions were compared to normal dorsal skin or tail lesions compared to tail skin. *Lgr5* expressing cells in hair follicles were recently shown to be targets of papillomavirus induced cancer [81] but there is not a clear indication by these results as to how this is involved with benign versus locally aggressive tumors. More detailed longitudinal studies are needed to resolve this point. Tumor suppressor genes (*Trp53* and *Trp63*), while statistically dysregulated, had a fold change of 1.3 or less. Similar results were seen with genes in the beta catenin (*Ctnnb1*), hedgehog (*Shh* and *Sufu*), smoothen (*Smo*), patched 1 (*Ptch1*) and related genes which are involved in a common canonical pathway with skin cancers, notably basal cell type tumors, in mice [82], [83]. It can be easy to interpret simple comparisons between tumors and normal skin from the same anatomical site in a highly controlled environment. However, when comparing a variety of test systems concurrently at different anatomic sites even when using a controlled method to induce cancer, in this case MmuPV1, interpretation becomes less straight forward and dogma disappears, as appears to be the case here.

These studies lay the ground work for investigating strain-specific modifier genes that show that some inbred strains of mice are susceptible to viral infection and tumor development, while others are not, a common observation with viral diseases [84]. It emphasizes the value of inbred strains which enable identification of these strain susceptibility differences [59], [60]. Many genetic mutations on the C57BL/6J and related substrains are now readily available to further refine the role of molecular differences between the skin at different anatomic sites. Therefore, finding anatomically different tumor types on the face or tail versus the dorsal truncal skin or vagina will help determine the differences in types of tumors, both malignant and benign. Our mouse model for papillomavirus infection provides the first opportunity to define mechanisms of this oncogenic virus at the cellular and molecular level.

## Materials and Methods

### Mice

Mice were obtained from The Jackson Laboratory (Bar Harbor, ME). Mice used were C57BL/6J (JR#664), NU/J-*Foxn1^nu^*/J (JR#2019), B6.Cg-*Foxn1^nu^*/J (JR#819), B6.129S7-*Rag1^1tm1Mom^/*J (JR#2216), NOD.129S7(B6)-*Rag1^1tm1Mom^/*J (JR#3729), NOD.CB17-*Prkdc^scid^*/J (JR#1303), B6.CB17-*Prkdc^scid^*/SzJ (JR#1913), B6.129S2-*Ighm^tm1Cgn^*/J (JR#2288), B6.129S2-*Cd4^tm1Mak^*/J (JR#2663), and B6.129S2-*Cd8^tm1Mak^*/J (JR#2665). Only female mice were used to minimize fighting. Mice were maintained in an humidity-, temperature-, and light cycle (12∶12) controlled vivarium under specific pathogen-free conditions (http://jaxmice.jax.org/html/health/quality_control.shtml#Animalhealth). Mice were housed in double-pen polycarbonate cages (330 cm^2^ floor area) at a maximum capacity of four mice per pen. Mice were allowed free access to autoclaved food (NIH 31, 6% fat; LabDiet 5K52, Purina Mills, St. Louis, MO) and acidified water (pH 2.8–3.2). All studies were done with The Jackson Laboratory Animal Care and Use Committee (approval number 11007) or the University of Louisville Animal Care and Use Committee (approval number 10141 and 10006) and Biosafety Committee (approval number IBC #10-024) approvals.

### MmuPV1 inoculum

Papillomas caused by MmuPV1 were either obtained from NMRI-*Foxn1^nu^/Foxn1^nu^* (nude) mice, from which MmuPV1 was originally isolated, or B6.Cg-*Foxn1^nu^/Foxn1^nu^* mice, in which experimental transmission studies were first conducted. The MmuPV1-infected papillomas were pulverized in liquid nitrogen with a mortar and pestle pre-cooled with dry-ice, suspended in Dulbecco’s phosphate-buffered saline (DPBS, Life Technologies, Carlsbad, CA), and stored at −80°C. The quantity of viruses in the inoculum was standardized for the L1 major capsid protein concentration, as identified by immunoblot, and the concentration calculated by band density on Coomassie Blue-stained SDS-PAGE gels compared with that of L1 protein of purified-MmuPV virus-like particles (VLP), as previously described in detail [26]. Band densities were measured using a molecular imager (PharosFx Plus; BioRad, Hercules, CA) and the Quantity One 4.5 program (BioRad). The total and L1 protein concentrations of inoculums used in this study were 10 µg and 0.3 µg per µl, respectively.

### Inoculation and Sample Collection

Prior to inoculation with the papilloma extract, all mice were anesthetized using tribromoethanol (400 mg/kg intraperitonealy, IP). Initially, to determine if the tumors contained infectious materials, 3 frozen tumors from different mice were utilized. Using a mortar and pestle, the samples were ground and then placed in a 15 ml centrifuge tube to which 2 ml of sterile phosphate buffered saline (PBS) was added. The mixture was pipetted to suspend and disperse the ground tissue, and then centrifuged at 1500 rpm 4°C, 5 min. The supernatant was aliquoted and stored at −80°C. Subsequently, 0.5 ml of papilloma extract was injected intradermally into two sites in the dorsal paralumbar fossa of four, 8 week old *B6.Cg-Foxn1^nu^/J* females and four, 8 week old NU/J- *Foxn1^nu^/Foxn1^nu^* female mice.

Scarification was determined to be a more efficient and used for subsequent studies. For cutaneous inoculation, small areas of skin (0.5 cm^2^ on the muzzle, dorsal back and/or tail) were scarified with a 20 gauge needle. One µl of the MmuPV1 cell-free homogenate was applied at each site. For maximum efficiency of infection, the scarified skin was then gently rubbed with the side of pipet tip. For the genital inoculation, 3 mg of medroxyprogesterone acetate (Depo Provera, Pfizer, New York, NY) were injected subcutaneously. After 3 days, 4 mg Nonoxynol-9 (Options Conceptrol Vaginal Contraceptive Gel, Caldwell Consumer Health**,** Madison, NJ) was injected into the vagina. Six hours later, 40 ∼100 ul of 10% MmuPV1, mixed with 0.1% carbopol-base gel (Carbopol 974P, glycerin, EDTA, methylparaben, and propylparaben, Magee Women's Research Institute, Pittsburgh PA), was inoculated into the vagina [85].

Mice were observed on a daily basis. When lesions approached 1 cm in diameter, in accordance with IACUC regulations, mice were euthanized and tumors were collected. Biopsies were bisected. One half was snap-frozen in liquid nitrogen and stored at −80°C for infection and molecular studies. The remaining tissue was fixed in Fekete’s acid-alcohol-formalin for histological studies. Blood was collected weekly by tail or submandibular venopuncture. At the time of euthanasia by CO_2_ asphyxiation mice were exsanguinated by open chest cardiac puncture. Blood was transferred to a microcontainer tube containing a serum separator (BD: Becton, Dickinson and Company, Franklin Lakes, NJ), centrifuged, and refrigerated at 4°C until used.

### Molecular testing for MmuPV1 by PCR

To extract and purify the DNA from biopsies, tissue samples were finely minced with scalpels and DNA was extracted using a kit (DNeasy blood and tissue kit; Qiagen; Valencia, CA) according to the manufacturer’s protocol. The concentration of DNA was measured using the Nanodrop ND-8000 (Thermo Scientific, Waltham, MA) at 260 nm.

For the PCR reactions, 3 µl of the DNA template at 0.1 µg/µl was added into 17 µl of PCR mixture (0.5 µM of MusPV-My09/11 primers [26], 0.5 mM of dNTP, 2 mM MgSO_4_, 1 unit of Platinum High Fidelity *Taq* polymerase (Life Technologies, Carlsbad, CA), 1X HiFi PCR buffer). Amplification was conducted by preheating for 1 min at 94°C followed by 45 cycles of 45 s at 94°C, 45 s at 57°C or 62°C, and 45 s at 68°C. The final extension was done for 10 min at 68°C. All samples were loaded onto a 1.5% agarose gel (UltraPure Agarose; Invitrogen) and a 1 Kb plus marker (Life Technologies, Carlsbad, CA) was used as a molecular standard. The primers were optimized to distinguish MmuPV1 from these other viral genomes as described and illustrated previously in detail [26].

### H&E staining, Immunohistochemistry

Tissues were fixed by immersion in Fekete’s acid-alcohol-formalin overnight, processed routinely, embedded in paraffin, sectioned at 6 um, and stained with hematoxylin and eosin (H&E). Serial sections were also tested for the presence of PV group-specific antigens by immunochemistry as previously described [24], [26] using rabbit polyclonal antibodies raised against disrupted *Canis familiaris* papillomavirus type 2 (CfPV2; previously designated as CPV2) virions. Antibodies reacting with the following markers were used on serial sections at concentrations as indicated on our public web page (http://tumor.informatics.jax.org/html/antibodies.html) [86] using a Ventana autostainer (Tuscon, AZ): rabbit anti-keratin 17 (KRT17) [87], kindly provided by P. Coulombe, rabbit anti-mouse KRT1 (stock# PRB-165P), KRT5 (stock# PRB-160P), KRT6A (stock# PRB-169P), KRT10 (stock# PRB-159P), KRT14 (stock# PRB-155P; Covance, Berkeley, CA), Ki-67 (RM-9106-R7 pre-diluted; Thermo Fisher Scientific, Waltham, MA) and SOAT1 (1∶200; stock# ab39327, Abcam, Cambridge MA). Diaminobenzidine (Sigma, St. Louis, MO) was used as chromogen. In addition, a rabbit-anti adipophilin/ADRP (1∶200, cat #GP40, Progren, Heidelberg, Germany) was used with the avidin-biotin method and the Bluemap kit (cat #760-120, Ventana).

### Affymetrix gene arrays

To determine if there were transcriptome differences between papillomas on the tail skin compared to invasive trichoblastomas on the dorsal skin, tumors from the tail and dorsal skin from 3 B6.Cg-*Foxn1^nu^/Foxn1^nu^* mice and unaffected skin from the tail or contra-lateral (dorsal skin) were collected and RNA extracted by The Jackson Laboratory Institutional Gene Expression Shared Service. Samples were tested using the Affymetrix *GeneChip Mouse Genome 1.0 ST Array* (Affymetrix, Santa Clara, CA). Concurrently, a matched study was done at the University of Louisville comparing facial (muzzle) papillomas and dorsal skin trichoblastomas to respective unaffected contralateral skin. The same procedures were used at both institutions and the statistical and pathway analyses were done at The Jackson Laboratory by the Computational Biology Department (TS).

Briefly, skin and tumor samples were stored in RNAlater (Ambion, Austin, TX) per the manufacturer’s instructions and homogenized in TRIzol (Life Technologies, Carlsbad, CA). Total RNA was isolated by standard TRIzol methods, and quality assessed using a 2100 Bioanalyzer instrument and RNA 6000 Nano LabChip assay (Agilent Technologies, Palo Alto, CA). Following reverse transcription with an oligo(dT)-T7 primer (Affymetrix, Santa Clara, CA), double-stranded cDNA was synthesized with the GeneChip Expression 3′–Amplification One-cycle kit (Affymetix).

In an *in vitro* transcription (IVT) reaction with T7 RNA polymerase, the cDNA was amplified and labeled with biotinylated nucleotides (Affymetrix). Ten micrograms of biotin-labeled and fragmented cDNAs were hybridized onto 1.0 ST GeneChip arrays (Affymetrix) for 16 hours at 45°C. Post-hybridization staining and washing was done according to the manufacturer's protocols using a Fluidics Station 450 instrument (Affymetrix). Finally, the arrays were scanned with a GeneChip Scanner 3000 laser confocal slide scanner. The images were quantified using GCOS 1.0 software (GeneChip Operating Software, Affymetrix).

Average signal intensities for each probe set within arrays were calculated by and exported from Affymetrix’s Expression Console (Version 1.2) software using the RMA method which incorporates convolution background correction, sketch-quantile normalization, and summarization based on a multi-array model fit robustly using the median polish algorithm. For this experiment, four pairwise comparisons were used to statistically resolve gene expression differences between sample groups using the R/maanova analysis package [88]. Specifically, differentially expressed genes were detected using Fs, a modified *F*-statistic incorporating shrinkage estimates of variance components from within the R/maanova package [88], [89]. Statistical significance levels of the pairwise comparisons were calculated by permutation analysis (1000 permutations) and adjusted for multiple testing using the false discovery rate (FDR), *q*-value, method [90]. Differentially expressed genes were declared at an FDR *q*-value threshold of 0.05.

Ingenuity pathway analysis software (IPA; Ingenuity Systems, www.ingenuity.com, Spring 2013 release) was used for network generation using a subset of overlapping differentially expressed genes identified to exist uniquely in 2 separate data sets (B6.Cg-*Foxn1^nu^*/J dorsal versus tail tumor tissues; B6.Cg-*Foxn1^nu^*/J dorsal versus facial tumor tissues). In total, eight microarray analyses were run across two experiments. The first experiment used four analyses in the comparison of skin and tumor tissues in both tail and dorsal regions. Analyses included: DL vs. DS, TL vs. TS, DL vs. TL, and DS vs. TS (D = dorsal, T = tail, L = lesion, and S = skin). The second experiment used another four analyses in the comparison of skin and tumor tissues in facial and dorsal regions. Analyses included: DL vs. DS, FL vs. FS, DL vs. FL, and DS vs. FS (F = Facial). To identify which set of genes were responsible for the differences in malignancy between lesion regions, we used the probe sets identified uniquely to DL vs. TL (1,792 genes) & DL vs. FL (524 genes). We used the probe sets overlapping in these two analyses for network analyses. The second set was generated independent of the first and analyzed concurrently. Data sets uploaded into IPA consisted of Affymetrix Mouse Gene 1.0 ST probeset IDs as identifiers. Each identifier was mapped to its corresponding object in the IPA knowledge base. These objects were overlaid onto a global gene network developed from information contained in the IPA Knowledge Base (Spring 2013 release). Expression results were limited to genes having a q-value<0.05. Networks of genes were algorithmically generated based on their connectivity for each of the comparison data sets. The top (2) networks generated for the unique subset of genes were retained. The dorsal vs. tail data set’s expression and significance values were overlaid onto the networks, so as to identify patterns of up- and down-regulated genes. In each network, molecules are represented as nodes, and the biological relationship between two nodes is represented as an edge (line). All edges are supported by at least one reference from the literature, from a textbook, or from canonical information stored in the IPA Knowledge Base. The intensity of the node color indicates the degree of up- (red) or down- (green) regulation with regards to all expression values in the data set. Nodes are displayed using various shapes that represent the functional class of the gene product. Edges are displayed with various labels that describe the nature of the relationship between the nodes.
